# Zidovudine and Interferon Alfa based regimens for the treatment of adult T-cell leukemia/lymphoma (ATLL): a systematic review and meta-analysis

**DOI:** 10.1186/s12985-023-02077-0

**Published:** 2023-06-07

**Authors:** Arman Shafiee, Niloofar Seighali, Nooshin Taherzadeh-ghahfarokhi, Shayan Mardi, Sorour Shojaeian, Shahrzad Shadabi, Mahsa Hasani, Sabahat Haghi, Sayed-Hamidreza Mozhgani

**Affiliations:** 1 Department of Psychiatry and Mental Health, Alborz University of Medical Sciences, Karaj, Iran; 2 Student Research Committee, School of Medicine, Alborz University of Medical Sciences, Karaj, Iran; 3 Department of Biochemistry, Medical Genetics, Nutrition, Alborz University of Medical Sciences, Karaj, Iran; 4 Department of Microbiology, School of Medicine, Alborz University of Medical Sciences, Karaj, Iran; 5 Department of Pediatrics, School of Medicine, Alborz University of Medical Sciences, Karaj, Iran; 6 Non-communicable Diseases Research Center, Alborz University of Medical, Karaj, Iran

**Keywords:** Adult T-Cell Leukemia/Lymphoma, Human T-cell leukemia virus type 1, Interferon Alfa, Zidovudine, Antiviral therapy

## Abstract

**Background:**

ATLL (Adult T-Cell Leukemia/Lymphoma) is an aggressive hematological malignancy. This T-cell non-Hodgkin lymphoma, caused by the human T-cell leukemia virus type 1 (HTLV-1), is challenging to treat. There is no known treatment for ATLL as of yet. However, it is recommended to use Zidovudine and Interferon Alfa-based regimens (AZT/IFN), chemotherapy, and stem cell transplant. This study aims to review the outcome of patients with different subtypes of ATLL treated with Zidovudine and Interferon Alfa-based regimens.

**Methods:**

A systematic search was carried out for articles evaluating outcomes of ATLL treatment by AZT/IFN agents on human subjects from January 1, 2004, until July 1, 2022. Researchers assessed all studies regarding the topic, followed by extracting the data. A random-effects model was used in the meta-analyses.

**Results:**

We obtained fifteen articles on the AZT/IFN treatment of 1101 ATLL patients. The response rate of the AZT/IFN regimen yielded an OR of 67% [95% CI: 0.50; 0.80], a CR of 33% [95% CI: 0.24; 0.44], and a PR of 31% [95% CI: 0.24; 0.39] among individuals who received this regimen at any point during their treatment. Our subgroup analyses’ findings demonstrated that patients who received front-line and combined AZT/IFN therapy responded better than those who received AZT/IFN alone. It is significant to note that patients with indolent subtypes of disease had considerably higher response rates than individuals with aggressive disease.

**Conclusion:**

IFN/AZT combined with chemotherapy regimens is an effective treatment for ATLL patients, and its use in the early stages of the disease may result in a greater response rate.

**Supplementary Information:**

The online version contains supplementary material available at 10.1186/s12985-023-02077-0.

## Introduction

Adult T-cell leukemia-lymphoma (ATLL) is a highly aggressive malignancy of mature T-lymphocytes with a generally poor prognosis. It occurs in only a small percentage (5–10%) of individuals infected with human T-lymphotropic virus type I (HTLV-1), with prevalence varying based on ethnic origin [1, 2]. According to Shimoyama criteria, ATLL is classified into four subtypes (acute, lymphoma, chronic, and smoldering). They are associated with different outcomes and need different therapeutic regimens tailored to the clinical symptoms [3].

ATLL is characterized by a combination of clinical manifestations, elevated serum LDH levels, hypercalcemia, and specific morphologic/immunophenotypic characteristics of malignant cells, along with confirmed HTLV-1 infection [4, 5]. A variety of clinical manifestations in ATLL patients is due to diverse complications of involved organs by ATLL cells, opportunistic infections, and hypercalcemia. These symptoms often contribute to the extremely high mortality of the disease. Patients with indolent ATLL may have no clinical manifestations and are only identified during health routine checkups and laboratory tests [6].

Treatment regimens for ATLL include watchful waiting until the disease progresses, multiagent chemotherapy, Zidovudine (AZT) and interferon-alpha (IFN-ɑ) therapy (AZT/IFN), and allogeneic hematopoietic stem cell transplantation (allo-HSCT). A meta-analysis of ATLL survival between 1995 and 2008 revealed that antiviral treatment with the combination of AZT and IFN was highly influential in the leukemic subtypes of ATLL and significantly improved long-term survival in patients [4, 7, 8]. However, new agents are developed for ATLL treatments and relapse prevention such as purine analogs, histone deacetylase inhibitors, arsenic/IFN combination, monoclonal antibodies including anti-CC chemokine receptor 4 (CCR4 mAb/Mogamulizumab/Moga) and toxin fusion proteins [6, 9, 10]. Currently, there is no definite standard of care for the treatment of ATLL patients. Therefore, in this study, we aim to assess the impact of AZT/IFN therapy on the treatment of ATLL patients by conducting a systematic review and meta-analysis of available evidence in this field since 2004.

## Methods

This study is reported based on the Preferred Reporting Items for Systematic Reviews and Meta-Analyses (PRISMA) guidelines. The protocol of our study is registered at Alborz University of Medical Sciences with the number IR.ABZUMS.REC.1399.322.

### Search strategy

A systematic search was carried out through MEDLINE/ PubMed, Embase, and Web of Science (WoS) databases (from January 1, 2004, until July 1, 2022) by two reviewers (AS, PM) independently on outcomes of ATLL patients treated with AZT/IFN agents. The search strategy was (((((((antiviral[Title/Abstract]) OR (anti-viral[Title/Abstract])) OR (zidovudine[Title/Abstract])) OR (AZT[Title/Abstract])) OR (interferon-alpha[Title/Abstract])) OR (IFN[Title/Abstract])) OR (AZT/IFN[Title/Abstract])) AND ((((((“Leukemia-Lymphoma, Adult T-Cell“[Mesh]) OR (Adult T-Cell Leukemia[Title/Abstract])) OR (Adult T-Cell Lymphoma[Title/Abstract])) OR (ATLL[Title/Abstract])))). The complete search strategy which has been used for the search is summarized in Table S1 Supplementary data.

### Study selection and data extraction

Randomized clinical trials, observational studies (cross-sectional, case–control, or cohort), case series/reports, conference abstracts, and editorials/letters were included. To acquire reliable articles, the following criteria were considered; (1) Articles must include patients diagnosed with ATLL, regardless of its type; (2) An AZT/IFN approach should be conducted in the treatment of at least a group of patients. EndNote reference management software was used for the study selection process and to manage the acquired articles. After duplicate removal, the title and abstract of the studies were evaluated based on the inclusion criteria. Eventually, the full texts were screened in detail. The selection process was done by two authors independently (AS, NS). The data extraction form containing age, gender, study type, sample size, treatment, diagnosis, and the relevant outcome has been filled out by two researchers independently (NT, PM). A third reviewer resolved disagreements (AS).

### Outcomes

Outcomes were defined as Complete response (CR); partial remission (PR); and overall response (OR) as defined by most of the included studies which followed the criterion defined by International Consensus Response Criteria published in JCO in 2009 [11].

### Quality assessment

Quality assessment was conducted by using the National Institutes of Health quality assessment checklist for observational cohorts. The checklist included 14 questions designed to assess the quality of each study. Each item’s ratings are yes, or no, and the final quality assessment score was the sum of the sub-items. Studies with 10 or more scores are rated as “Good”, 7–9 scores as “Fair,“ and fewer than 7 are rated as “Poor”. The quality assessment was carried out by two researchers independently addressing the items reported in the guidelines (NS, NT).

### Statistical analysis

A proportion meta-analysis was carried out to estimate OR, CR, and PR of ATLL patients treated with AZT/IFN regimens. To normalize the data provided by each study, the study estimates were first logit-transformed. As study populations and methods varied across studies, a random effects model was used to summarize the response rate using proportions and 95% CIs. Heterogeneity was assessed using the Cochrane Q-test for heterogeneity and I^2^ statistic. I^2^ values of 25%, 50%, and 75% were considered low, moderate, and high amounts of heterogeneity, respectively. I^2^ less than 25% was considered to indicate low heterogeneity. Under this condition, a fixed-effects model was applied. Publication bias was assessed for relevant outcomes with at least 10 included studies using visual inspection of funnel plots and Egger’s regression test. Subgroup analyses were performed to assess the efficacy under these circumstances: (1) AZT/IFN regimen alone; (2) AZT/IFN used in front-line regimens; (3) AZT/IFN used for treating aggressive ATLL (acute, lymphomatous); and (4) AZT/IFN used for treating indolent ATLL (chronic, smoldering). All statistical analyses and graphics were carried out using R (version 4.1.3) (R Core Team, 2020) and the meta package.

## Results

### Characteristics of included studies

**Fig. 1:**
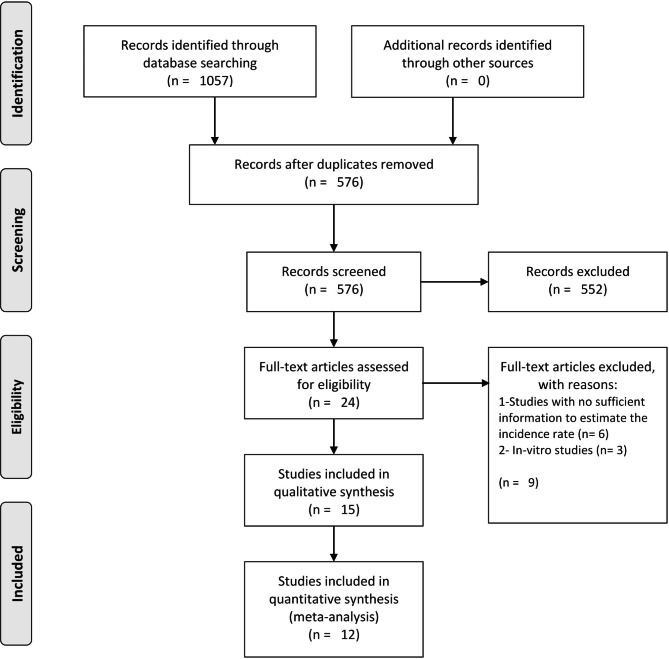
Study Flowchart

Our search strategy revealed 1057 articles and 481 duplicates were removed after screening by title and abstract. Based on inclusion criteria, 24 records were retrieved for evaluating the full text. Ultimately, we obtained 15 reports regarding the treatment of ATLL, which were performed between 2004 and 2022 (Fig. 1) [12–26]. Five of them were conference abstracts. Seven were raised in endemic countries, including South America and the Middle East. Table 1 shows the characteristics of the included studies and the quality assessment of results.

**Table 1:** Characteristics of included studies and quality assessment results

Author	Year	Journal/Conference abstract	Type	Sample Size	Country	Age	Treatment group	Diagnosis	Survival rate	Quality
Malpica	2022	Leukemia & Lymphoma	Retrospective cohort	169	Six centers from four Latin American countries (Argentina, Chile, Colombia, and Peru)	57	1. AZT-IFN alone 2. Multi-agent chemotherapy alone 3. Combination chemotherapy and AZT-IFN 4. Single-agent chemotherapy and/or regional therapy	Acute n = 54; lymphomatous n = 84; chronic n = 18; smoldering n = 5	-No report of survival rate based on the type of regimen	Good
Malpica	2021	JCO Global Oncology	Retrospective cohort	253	Eleven Latin American countries	57	1. AZT-IFN alone 2. Multi-agent chemotherapy alone 3. Combination chemotherapy and AZT-IFN 4. Single-agent chemotherapy and/or regional therapy	Acute n = 73; lymphomatous n = 122; chronic n = 26; smoldering n = 5	-In patients with aggressive ATLL who achieved complete response (CR), first-line treatment with AZT-IFN (alone or in combination with chemotherapy) showed better PFS compared to chemotherapy alone in acute ATLL. -The differences in PFS between the treatment approaches were not statistically significant.	Good
Guery	2021	Annals of Hematology	Retrospective cohort	47	France	51	1. Zidovudine-interferon alfa 2. Chemotherapy	Acute n = 23; lymphomatous n = 14; chronic n = 8; smoldering n = 2	-No report of survival rate based on the type of regimen.	Good
Nogueira	2020	Blood	Ambispective observational study	41	Brazil	50	1. chemotherapy with anthracycline-based regimens 2. immunotherapy and antiviral therapy	Acute 29%; lymphomatous 46%; chronic 17%; smoldering 8%	-No report of survival rate based on the type of regimen.	Poor
Malpica	2018	Blood	Retrospective cohort	195	USA	52	1. chemotherapy alone, 2. combined chemotherapy with AZT-IFN (concurrently or sequentially), 3. AZT-IFN alone.	Acute n = 80; lymphomatous n = 96; chronic n = 5; unfavorable chronic n = 7; smoldering n = 3	-In patients with aggressive adult T-cell leukemia/lymphoma (ATLL) who achieved complete response (CR) after treatment, the median progression-free survival (PFS) was 48 months for those who received AZT-IFN. -In contrast, patients who achieved CR after chemotherapy had a median PFS of 11 months. -The difference in PFS between AZT-IFN and chemotherapy was statistically significant (p = 0.003).	Good
Oliveira	2017	Brazilian Journal of Hematology and Hemotherapy	Retrospective cohort	83	Brazil	NR	1. first-line multiagent chemotherapy, 2. first-line antiviral therapy, 3. chemotherapy associated with antiviral therapy	Acute n = 16; lymphomatous n = 13; chronic n = 23; smoldering n = 26; primary cutaneous tumoral n = 5	-Favorable chronic patients treated with antivirals had longer survival compared to the unfavorable subtype. -In the case of the acute form of the disease, first-line chemotherapy showed better survival outcomes compared to antivirals, although the difference was not statistically significant.	Good
Zell	2016	Oncotarget	Retrospective cohort	53	USA	54	1. Chemotherapy Only, 2. Chemotherapy with antiviral	Acute n = 36; lymphomatous n = 14; chronic/smoldering n = 3	-There was no significant difference in survival between patients who received chemotherapy alone and those who received chemotherapy with antiviral agents.	Good
Cordeiro	2015	Blood	Retrospective cohort	29	Brazil	49	1. Interferon alpha and zidovudine (IFN + AZT), 2. Polychemotherapy, usually with CHOEP regimen	Acute n = 15; lymphomatous n = 3; chronic n = 6; smoldering n = 5	-Patients with acute adult T-cell leukemia/lymphoma (ATLL) treated with chemotherapy as first-line therapy had a median overall survival (OS) of 5.8 months. -This OS result is comparable to the average survival of 6 months reported in the literature for acute ATLL patients treated with chemotherapy. -However, patients treated with first-line IFN + AZT (interferon + azidothymidine) had a longer average survival of 9 months compared to chemotherapy alone.	Fair
Pimentel	2014	Retrovirology	Retrospective cohort	108	USA	NR	1. High-dose AZT/interferon (IFN) as first line therapy, 2. Chemotherapy-based regimens	Acute n = 51; lymphomatous n = 50; chronic n = 5; smoldering n = 2	-In the study, several long-sustained responses were observed in patients with acute and unfavorable chronic subtypes of the disease. -These patients were treated with first-line AZT and IFN alone. -These sustained responses translated into a survival benefit for the patients.	Fair
Hodson	2014	Retrovirology	Case series	4	UK	52	First line treatment with zidovudine and interferon alpha (ZDV/IFN-a	Chronic n = 4	-All patients included in the study remained alive. -The median overall survival for these patients was 64 months, with a range of 27 to 106 months.	Fair
Fields	2014	Blood	Case series	3	UK	NR	1. Zidovudine (ZDV)/Interferon-a (IFN), anti-CD25/bortezomib; 2. ZDV/INF, anti-CD25/bortezomib, etoposide, Gemcitabine/Oxaloplatin; 3. Sodium valproate, ZDV/IFN, etoposide	Chronic n = 3	-No report of survival rate based on the type of regimen.	Poor
Kchour	2013	Retrovirology	Retrospective cohort	16	Iran	NR	Arsenic/IFN/zidovudine	Acute n = 2; lymphomatous n = 2; Chronic n = 12	-No report of survival rate based on the type of regimen.	Fair
Hodson	2011	Journal of Clinical Oncology	Retrospective cohort	73	UK	58	1. chemotherapy only 2. Antiviral treatment administered concurrently with or immediately sequentially to first-line chemotherapy 3. Initial chemotherapy with antiviral treatment administered at any time after relapse	Acute n = 29; lymphomatous n = 44	-The use of ZDV (zidovudine) and IFN (interferon) at any time resulted in prolonged survival in acute and lymphoma subtypes of adult T-cell leukemia/lymphoma (ATLL). -The use of ZDV/IFN was associated with a significant reduction in the risk of death in aggressive ATLL. -The hazard ratio for the reduction in risk of death was 0.23, with a 95% confidence interval of 0.09 to 0.60. -The use of ZDV/IFN in aggressive ATLL showed a significant association with improved survival (P = 0.002).	Good
Kchour	2007	Leukemia & Lymphoma	Observational cohort	20	Iran	51	AZT/IFN	Acute n = 9; lymphomatous n = 5; Chronic n = 14	-The study confirmed that treatment with AZT (azidothymidine) and IFN (interferon) induces a high response rate in patients. -This treatment also resulted in prolonged survival. -Importantly, the AZT/IFN treatment was associated with minimal side effects.	Fair
Hermine	2004	The Hematology Journal	Phase II trial	7	France	NR	Arsenic/IFN/zidovudine	Acute n = 4; lymphomatous n = 3	-No report of survival rate based on the type of regimen.	Poor

### Patients’ characteristics

A total number of 1101 patients were identified (acute: n = 404, lymphomatous: n = 469, chronic: n = 134, and smoldering: n = 94). The most common diseases among these patients were acute and lymphomatous. Most often, AZT/IFN was given along with chemotherapy. The median age of the study participants was 49 to 58 years.

### Quality assessment

Based on the results of our quality assessment, there were 7 studies with good, 5 with fair and 3 with poor methodological quality. Most of the included studies were observational, so they may be biased due to their retrospective design. All studies did not state if they blinded the outcome assessors to the exposure status of participants. It may bias the results by including unreliable data. Some studies did not clearly define exposure/outcome measures, so the exposure/outcome may not be valid and reliable. All five included studies were in the form of conference abstracts. Therefore, they didn’t provide sufficient details. For instance, the lack of well-defined, pre-specified inclusion and exclusion criteria for the study population was a common issue in conference abstracts. It was not clear in some studies if all eligible people had participated in the study, so it raised concerns about selection bias.

### Overall response in patients receiving AZT/IFN-based regimens

**Fig. 2:**
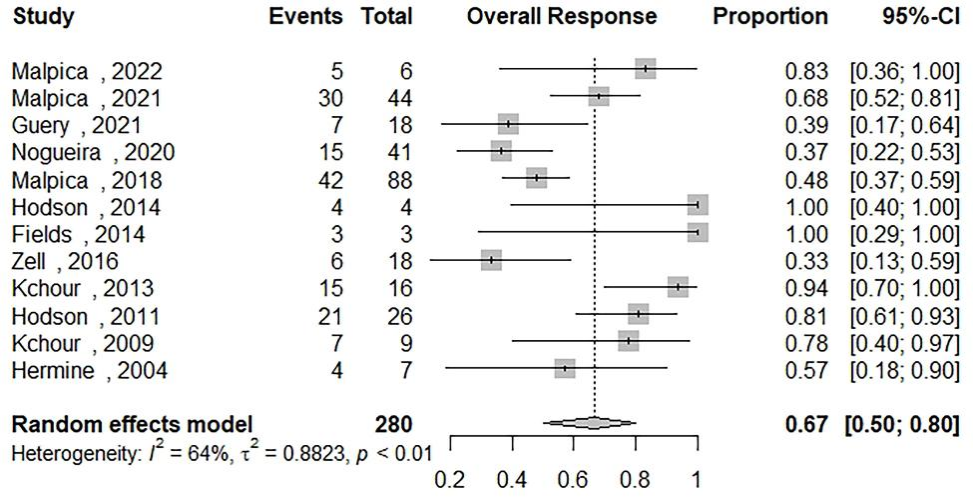
The pooled estimate of the overall response rate (OR) of ATLL patients receiving an Zidovudine and Interferon Alfa based regimens (AZT/IFN) during their therapeutic intervention

**Fig. 3:**
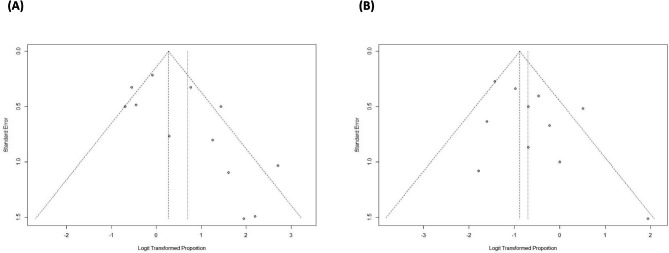
Funnel plot of overall response rate (OR) and complete response rate (CR) for evaluation of publication bias

Data on the overall response rate (OR) of ATLL patients who received AZT/IFN-based regimens were synthesized from 12 studies and the results were included in Fig. 2 [12–16, 18, 21–26]. Two hundred eighty out of 1101 patients were included. The overall response of AZT/IFN-contained regimens for all subtypes was 67% [95% CI: 0.50; 0.80] with moderate heterogeneity (Q = 30.38, I2 = 63.8%). We have performed subgroup analyses to determine the OR among patients who received (Table 2) : (1) AZT/IFN regimen without any combination therapy and the results showed an OR of 47% [95% CI: 0.25; 0.69, Q = 19.44, I2 = 74.3%]; (2) AZT/IFN used in front-line regimens showed an OR of 58% [95% CI: 0.45; 0.70, Q = 28.69, I2 = 61.7%]; (3) AZT/IFN used for treating aggressive ATLL (acute, lymphomatous) showed an OR of 68% [95% CI: 0.52; 0.80, Q = 17.29, I2 = 53.7%]; and (4) AZT/IFN used for treating indolent ATLL (chronic, smoldering) showed an OR of 86% [95% CI: 0.71; 0.94, Q = 0.00, I2 = 0.0%]. There was no publication bias based on the inspection of the funnel plot and Egger’s regression test (p-value = 0.06) (Fig. 3-(A)).

**Table 2:** Subgroup analyses of antiviral treatment for ATLL.

		Response Rate, Proportion [95% CI]	
Treatment	OR	CR	PR
AZT-IFN alone	0.47 [0.25; 0.69]	0.41 [0.20; 0.66]	0.25 [0.18; 0.33]
AZT-IFN used in front-line regimens	0.68 [0.52; 0.80]	0.34 [0.25; 0.44]	0.36 [0.30; 0.43]
AZT-IFN used for treating aggressive ATLL (acute, lymphomatous)	0.58 [0.45; 0.70]	0.25 [0.20; 0.31]	0.32 [0.26; 0.39]
AZT-IFN used for treating indolent ATLL (chronic, smoldering)	0.86 [0.71; 0.94]	0.53 [0.28; 0.76]	0.37 [0.22; 0.54]

OR: Overall response, CR: Complete response, PR: Partial response

### Complete response in patients receiving AZT/IFN-based regimens

**Fig. 4:**
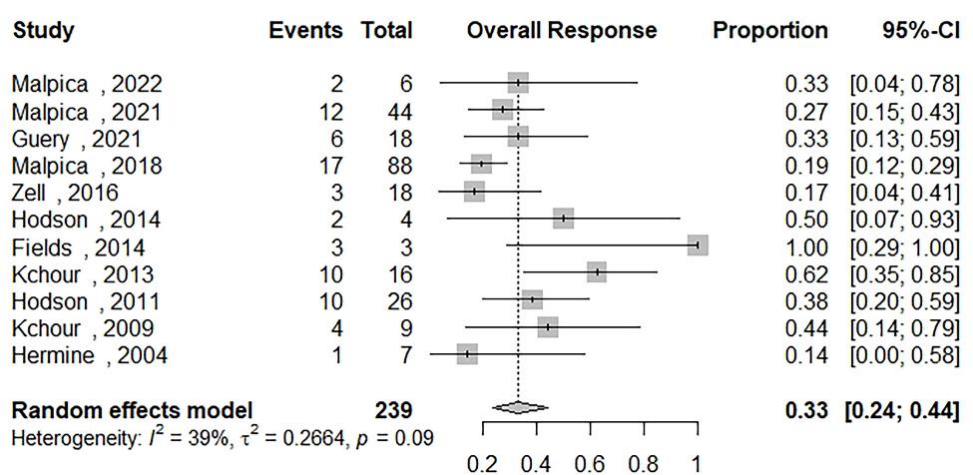
The pooled estimate of the complete response rate (CR) of ATLL patients receiving an Zidovudine and Interferon Alfa based regimens (AZT/IFN) during their therapeutic intervention

Data on the complete response rate (CR) of ATLL patients who received AZT/IFN-based regimens were synthesized from 11 studies and the results were included in Fig. 4 [12–14, 16, 18, 21–26]. Two hundred thirty-nine out of 1101 patients were included. The complete response of AZT/IFN-contained regimens for all subtypes was 33% [95% CI: 0.24; 0.44] with moderate heterogeneity (Q = 16.48, I2 = 39.3%). We have performed subgroup analyses to determine the CR among patients who received : (1) AZT/IFN regimen without any combination therapy and the results showed a CR of 41% [95% CI: 0.20; 0.66, Q = 7.78, I2 = 35.8%]; (2) AZT/IFN used in front-line regimens showed a CR of 34% [95% CI: 0.25; 0.44, Q = 13.76, I2 = 27.3%]; (3) AZT/IFN used for treating aggressive ATLL (acute, lymphomatous) showed a CR of 25% [95% CI: 0.20; 0.31, Q = 6.15, I2 = 0.0%]; and (4) AZT/IFN used for treating indolent ATLL (chronic, smoldering) showed a CR of 53% [95% CI: 0.28; 0.76, Q = 6.71, I2 = 25.4%]. There was no publication bias based on the inspection of the funnel plot and Egger’s regression test (p-value = 0.14) (Fig. 3-(B)).

### Partial response in patients receiving AZT/IFN-based regimens

**Fig. 5:**
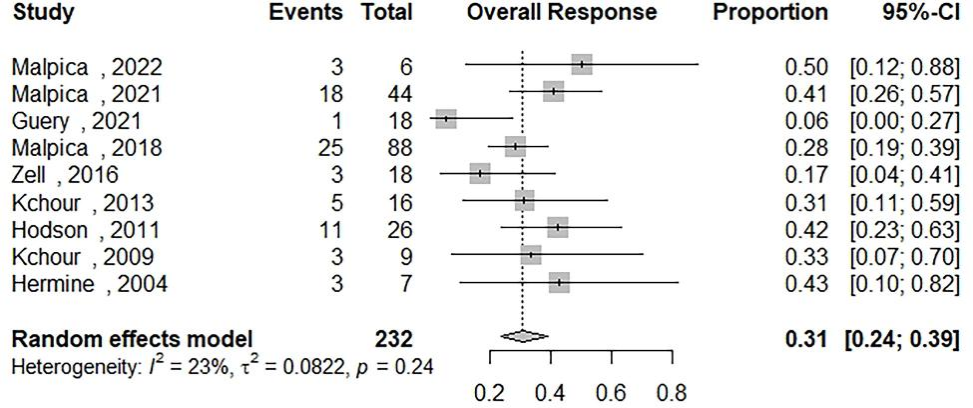
The pooled estimate of the partial response rate (PR) of ATLL patients receiving an Zidovudine and Interferon Alfa based regimens (AZT/IFN) during their therapeutic intervention

Data on the partial response rate (PR) of ATLL patients who received AZT/IFN-based regimens were synthesized from 9 studies and the results were included in Fig. 5 [12–14, 16, 18, 23–26]. Two hundred thirty-two out of 1101 patients were included. The partial response of AZT/IFN-contained regimens for all subtypes was 31% [95% CI: 0.24; 0.39] with low heterogeneity (Q = 10.44, I2 = 23.4%). We have performed subgroup analyses to determine the OR among patients who received : (1) AZT/IFN regimen without any combination therapy and the results showed a PR of 25% [95% CI: 0.18; 0.33, Q = 0.48, I2 = 0.0%]; (2) AZT/IFN used in front-line regimens showed a PR of 36% [95% CI: 0.30; 0.43, Q = 4.40, I2 = 0.0%]; (3) AZT/IFN used for treating aggressive ATLL (acute, lymphomatous) showed a PR of 32% [95% CI: 0.26; 0.39, Q = 9.82, I2 = 18.5%]; and (4) AZT/IFN used for treating indolent ATLL (chronic, smoldering) showed a PR of 37% [95% CI: 0.22; 0.54, Q = 2.01, I2 = 0.0%]. Amount of studies (k = 9) was too small to examine the publication bias.

## Discussion

In this study, we present the latest findings on the utilization of the AZT/IFN regimen for treating ATLL patients. Our analysis included fifteen studies, primarily retrospective cohorts, encompassing a total of 1101 ATLL patients. The majority of the available treatment data focused on patients with the aggressive forms of the disease, namely the acute and lymphomatous subtypes.

Our quantitative analysis of the AZT/IFN regimen’s response rate revealed an OR of 67%. Among individuals who received this regimen at any point during their treatment, a complete response (CR) rate of 33% and a partial response (PR) rate of 31% were observed. Subgroup analyses demonstrated that patients receiving front-line and combined AZT/IFN therapy exhibited higher response rates compared to those receiving AZT/IFN alone. Notably, patients with indolent subtypes showed significantly higher response rates compared to those with aggressive diseases.

While these findings suggest the potential benefits of initiating AZT/IFN-containing regimens early in the disease course, it is important to note that more research with an adequate sample size of indolent patients is needed to precisely investigate the effect of the AZT/IFN regimen in the early stages of ATLL.

The combination of AZT (azidothymidine) and IFN-α (alpha-interferon) was initially proposed by Gill et al.[27] and Hermine et al. [28] and is currently recognized as one of the most effective therapies for ATLL [7]. his combination is believed to suppress ATLL cells by activating the immune response against HTLV-1-infected cells and interfering with HTLV-1 replication [29]. Zidovudine, an anti-HIV drug, can also inhibit HTLV-1 transmission, and when combined with alpha-interferon, a naturally produced cytokine that responds to viral infections, it has shown positive effects in ATLL treatment. Additionally, the presence of wild-type p53 has been associated with a better response to AZT treatment [27]. Arsenic trioxide is another antiviral that impedes the growth of HTLV-1 and induces apoptosis [30]. Current data cannot evaluate the synergic effect of the chemotherapy and AZT *plus* IFN-α *plus* Arsenic effect. However, more research is needed to demonstrate that these regimens are attributed to better clinical response and longer medial survival time (MST) than chemotherapy. The pathophysiology of these regimens is described in Table 3.

**Table 3:** Mechanistic basis of treatments used for the treatment of ATLL.

Treatment	Pathophysiology
IFN	• Preventing targeting of viral Gag proteins to the rafts in the plasma membrane
	• Induction of 2′-5′oligoadenylate synthase and protein kinase P1
	• Stimulation of natural killer cells and macrophages and enhancing antigen presentation to lymphocytes.
	• Activation of Jak1 and Tyk2, which lead to induction of tyrosine phosphorylation of the eIF2alpha kinase PKR
AZT	• Inhibition of the reverse transcriptase of the HTLV-1 virus
	• Inhibition of telomerase which results in progressive telomere shortening and activation and stabilization of TTP53 in ATLL cases
IFN + ARS	• A rapid shutdown of the NF-kappa B pathway results in the induction of cell cycle arrest and apoptosis
	• Restoring PML nuclear body formation
Mogamulizumab	• Targeting CCR4, which is a chemokine receptor that is preferentially expressed by Th2 and regulatory T cells which lead to promoting T-cell migration

HTLV-1 genome includes several genes such as tax and HBZ. Tax stimulates the proliferation of T-lymphocytes and inhibits their apoptosis by activating pathways including the NF-kappa B signaling pathway, Basal transcription factors, actin cytoskeleton, P53 pathway, and PI3K-Akt signaling pathway[31, 32]. Although Tax proliferation is vital for the expansion of the infected CD4 + T cells, it provokes the immune system and may decline the number of these cells. Genetic and epigenetic alternations, mostly unknown, accumulate during the latent period and result in the development of these cells to ATLL [33]. These alternations lead to different types of ATLL ranging from smoldering ATLL, which is usually asymptomatic or presents with skin rashes, to chronic type, characterized by lymphocytosis and is stable for months or even years, and acute and lymphoma type, which manifests systemic symptoms and progress quickly [34].

Bazarbachi et al. [7] conducted a meta-analysis in 2010 that aimed to review AZT/IFN effects on ATLL treatment in trials. Their study, similar to our systematic review, concluded that first-line AZT/IFN therapy leads to a better survival rate than chemotherapy alone. Since 2010, some studies extended our knowledge of clinical outcomes of the combination of AZT/IFN; for example, Datta et al. study demonstrated that this combination has a significant effect on patients with wild type p53 however it is not helpful in the treatment of patients with mutated inactive p53 [35]. Furthermore, Oliveira et al. showed that the MST was longer in patients with the chronic type who received AZT and IFN on their first-line therapy (44 months). At the same time, MST of patients with acute form was conversely longer in the group of patients who got first-line chemotherapy than the group which got both chemotherapy and AZT/IFN-α. Among all types, the smoldering form has the longest MST. Moreover, lymphoma and acute type have shorter MST than chronic types, despite the method of treatment[17]. Kchour et al. study added Arsenic (As) to the combination of AZT and IFN. Their triple regimen that consists of As/IFN/AZT is attributed to an impressive response in ATLL patients, especially in chronic type. That is to say, the complete response rate is lower in patients who get AZT and IFN alone in comparison to patients who receive a triple regimen [36]. In 2017, Oliveira et al. evaluated the mean survival time (MST) for the antiviral and chemotropic regimen [17]. The highest MST was among smoldering-type patients who received skin-directed therapy and watchful waiting, whereas the participants with different ATLL subtypes treated with VCAP-AMP-VECP (vincristine, cyclophosphamide, doxorubicin and prednisolone; doxorubicin, ranimustine and prednisolone; vindesine, etoposide, carboplatin and prednisolone) had the lowest MST. On average, patients treated with AZT *plus* IFN-α lived 11.35 months longer than patients treated with VCAP-AMP-VECP.

Apart from chemotherapy, and stem cell transplantation, which approach ATLL treatment as a malignancy therapy, some therapeutic approaches toward ATLL consider the disease a complication of a viral infection with HTLV-1. For instance, Mogamulizumab, a monoclonal antibody targeting the CCR-4 receptor, causes a reduction in the number of HTLV-1 infected cells. Several studies revealed that Mogamulizumab has a great response in patients diagnosed with an acute type of ATLL [37–39]. One of the highest complete response rates (100%) was found in Kawano et al. study, which used Mogamulizumab *plus* Allo-HSCT *plus* VCAP-AMP-VECP for treatment. Meanwhile, Mogamulizumab *plus* MLSG15 had a relatively lower complete response rate (37.5%). (MLSG15 regimen is a dose-intensified multidrug regimen, namely the modified LSG15 (mLSG15) regimen including VCAP‐AMP‐VECP). In addition, the combination of MLSG15 and Mogamulizumab had a higher PR rate (41%) and CRU rate (25%) than MLSG15 alone (34%). It should be noted that Mogamulizumab *plus* Allo-HSCT *plus* CHOP regimen was not effective in lymphoma Type (CR = 0%). Ishida et al. study indicated that people treated with mLSG15 *plus* Mogamulizumab (52%) have a higher complete remission rate than patients who received mLSG15 alone (33%). [38]. In addition, Kawano et al. showed that using Mogamulizumab before allogeneic-HSCT increases the survival time. They can be used in the refractory type of diseases, although one of the disadvantages of this treatment is the increased chance of severe GVHD (graft versus host diseases reaction). One way to solve this problem is to increase the interval between the last Mogamulizumab administration and allo-HSCT [37].

Our study faced several limitations. First, there were not so many articles that reported the clinical outcome of ATLL patients who received the AZT/IFN regimen. Second, there was a paucity of data because several studies included in our meta-analysis were conference abstracts. Third, the conclusions were made based on a limited number of observational studies, which lowers the certainty of evidence because of their retrospective nature. Finally, the possible variation in the outcome criterion among the studies could be contributed to the heterogeneity seen in the results of our meta-analysis. One of the major concerns regarding AZT/IFN-based regimens is the discontinuation of the production of intravenous and subcutaneous interferon alfa-2b. As of September 2021, Merck & Co. has discontinued the production of both intravenous and subcutaneous formulations of interferon alfa-2b. The reasons for this decision have not been publicly disclosed. But there has been news regarding a business decision [40].

In conclusion, this study showed that prescribing AZT/IFN regimen is attributed to a favorable clinical response rate in ATLL patients. These findings indicate that, as a clinician, choosing an AZT/IFN therapy not merely depends on the patient’s circumstances, such as their malignancy type but also hangs on the treatments that patients have received. AZT/IFN combined with chemotherapy regimens is an effective treatment for ATLL patients, and its use in the early stages of the disease may result in a greater response rate. Future studies with vigourous foucus on the survival rate of the patients receiving these treatment is highly suggested to inform the clinicians regarding the efficacy of these regimens.

## Electronic supplementary material

Below is the link to the electronic supplementary material.

Supplementary Material 1

## Data Availability

All relevant data are within the paper.
