# Supplementary material for: Zidovudine and Interferon Alfa based regimens for the treatment of adult T-cell leukemia/lymphoma (ATLL): a systematic review and meta-analysis
Source: Virol J. 2023 Jun 7;20:118. doi: 10.1186/s12985-023-02077-0 (PMC10249295; doi:10.1186/s12985-023-02077-0)
Supplement: Supplementary file 1 — Supplementary Material 1 [file 12985_2023_2077_MOESM1_ESM.docx]

**Supplementary data**

**Table S1: Databases searched and search strategies employed – PUBMED: 323,EMBASE: 404-after dup:**

| **Database** | **Search strategy** |
| --- | --- |
| PubMed | (((((((antiviral[Title/Abstract]) OR (anti-viral[Title/Abstract])) OR (zidovudine[Title/Abstract])) OR (AZT[Title/Abstract])) OR (interferon-alpha[Title/Abstract])) OR (IFN[Title/Abstract])) OR (AZT/IFN[Title/Abstract])) AND (((((("Leukemia-Lymphoma, Adult T-Cell"[Mesh]) OR (Adult T-Cell Leukemia[Title/Abstract])) OR (Adult T-Cell Lymphoma[Title/Abstract])) OR (ATLL[Title/Abstract])))) |
| EMBASE | ('adult t-cell leukemia':ab,ti OR 'adult t-cell lymphoma':ab,ti OR atll:ab,ti) AND (antiviral:ab,ti OR 'anti viral':ab,ti OR zidovudine:ab,ti OR azt:ab,ti OR 'interferon alpha':ab,ti OR ifn:ab,ti) |
